# World Day of Remembrance for Road Traffic Victims — November 17, 2013

**Published:** 2013-11-15

Road traffic crashes kill nearly 3,500 persons each day and injure or disable 50 million each year around the world (1). Road traffic crashes are the leading cause of death among persons aged 10–24 years worldwide and the leading cause of death among those in the first 3 decades of life in the United States. CDC has declared road traffic injuries a “winnable battle” and supports efforts at the United Nations (UN) and World Health Organization (WHO) to dedicate 2011–2020 as the Decade of Action for Road Safety (2).

The Decade of Action was launched in May 2011 in approximately 100 countries, with the goal of preventing 5 million road traffic deaths globally by 2020. In October 2005, the UN General Assembly adopted a resolution calling for governments and nongovernmental organizations to mark the third Sunday in November each year as World Day of Remembrance for Road Traffic Victims (3). The observance was created to recognize persons injured or killed in road traffic crashes and the plight of relatives and others who must cope with the emotional and practical consequences of these events.

CDC, WHO, and the UN Road Safety Collaboration encourage governments and nongovernmental organizations worldwide to commemorate November 17, 2013, as the World Day of Remembrance to draw the public’s attention to road traffic crashes, their consequences and costs, and prevention measures. The theme of this year’s observance is “From Global Remembrance to Global Action Across the Decade.” Ancillary materials are available to provide organizations with action strategies to support victims and survivors (4). Practical guidance for persons or groups on how to plan and organize events on this day is available from WHO at http://whqlibdoc.who.int/publications/2006/9241594527_eng.pdf.

Additional information about the World Day of Remembrance is available at http://www.worlddayofremembrance.org. Additional information about CDC’s motor vehicle injury prevention activities is available at http://www.cdc.gov/winnablebattles/motorvehicleinjury.
